# *In vitro* assembly complex formation of TRAIP CC and RAP 80 zinc finger motif revealed by our study

**DOI:** 10.1016/j.sjbs.2021.08.083

**Published:** 2021-08-30

**Authors:** Eijaz Ahmed Bhat, Nasreena Sajjad, Irfan A. Rather, Jamal S.M. Sabir, Yan-Yan Hor

**Affiliations:** a Life Sciences Institute, Zhejiang University, Hangzhou, Zhejiang 310058, PR China; b Department of Biological Sciences and Bioengineering, Indian Institute of Technology, Kanpur 208016, India; c Department of Biochemistry, University of Kashmir, Hazratbal, Jammu and Kashmir, India; d Department of Biological Sciences, Faculty of Science, King Abdulaziz University, Jeddah 21589, Saudi Arabia; e Department of Biotechnology, Yeungnam University, 280 Daehak-Ro, Gyeongsan, Gyeongbuk 38541, Republic of Korea

**Keywords:** TNF-protein, RAP- 80, Protein interaction, NF-kB

## Abstract

**Background:**

Tumor necrosis factor interacting protein (TRAIP/TRIP) is an important cell-signaling molecule that prevents the TNF-induced-nuclear factor kappa-light-chain-enhancer of activated B cells (NF-κB) activation *via* direct interaction with TRAF 2 protein. TRAIP is a crucial downstream signaling molecule, implicated in several signaling pathways. Due to these multifunctional effects, TRAIP is more related to cellular mitosis, chromosome segregation, and DNA damage response. Tumor necrosis factor interacting protein is a downstream signaling molecule that contains a RING domain with E3 ubiquitin ligase activity at the N terminal side followed by coiled-coil and C terminal leucine zipper domain. Human TRAIP is constituted of 469 amino acids with 76% sequence similarity with the mouse TRAIP protein. Although, the main inhibitory function of TRAIP has been known for decades, however, *in vitro* interaction of TRAIPCC domain with RAP80 Zinc finger motif has not been reported yet. Besides, RAP80, the binding partner of TRAIPCC protein has been implicated in DNA damage response.

**Results:**

Our *in vitro* study shows that the TRAIP CC (64–166) associates with the RAP80 zinc finger of corresponding amino acid 490–584. However, TRAIP CCLZ (66–260) and TRAIP RINGCC (1 = 157) failed to interact with the RAP80 zinc finger of corresponding amino acid 490–584. The current study reinforces TRAIP CC (64–166) and RAP80 zinc finger of corresponding amino acid 490–584 associates to form a complex. Moreover, SDS PAGE arbitrated the homogeneity of RAP80 Zinc finger and TRAIP CC of corresponding amino acid 490–584 and 64–166, respectively.

**Conclusion:**

*In vitro*, a specific interaction was observed between the TRAIP CC (64–166) and the RAP80 zinc finger of the corresponding amino acid 490–584 and a specific binding area of the RAP80 zinc finger motif were investigated. The TRAIPCC region is required for the complex to bind to the RAP80-Zn finger motif. This strategy may be necessary for the RAP80 zinc finger activity to the TRAIP CC protein.

## Graphical abstract

**Figure:**
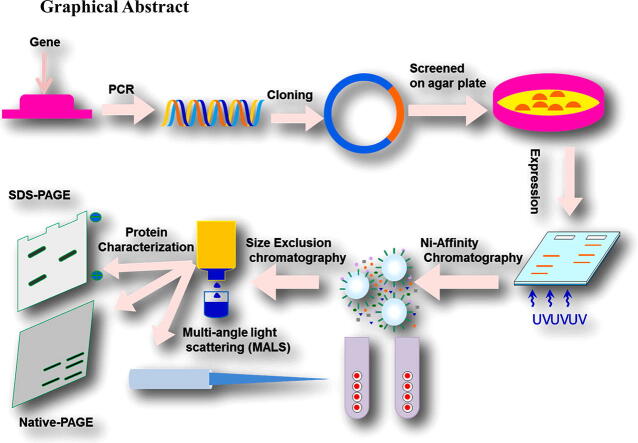

## 1 Introduction

TRAF-interacting protein (TRAIP) emerged as an important signaling molecule and negatively regulated TNF-induced nuclear factor (NF)-kB activation (Almeida et al., 2011). The TRAIP protein is composed of three domains with 469 amino acids, the N-terminal Ring domain, coiled-coil and C-terminal Leucine zipper domain (Almeida et al., 2011, Bartek et al., 2007). TRAIP plays a critical role in the conscription of RAP80 to DNA lesions and is a significant factor in other signaling processes (Besse et al., 2007). The main inhibitory function of TRAIP with direct interaction with TRAF2 and possibly TRAF1 has been reported by a previous study (Bhat et al., 2020, Bhat et al., 2018). Besides the main function, various other crucial functions of TRAIP, including cell proliferation, DNA damage response, antiviral response and mitotic progression of chromosomal segregation, have been reported (Bhat and Rather, 2018, Chapard et al., 2012, Harley et al., 2016, Hoffmann et al., 2016). Considering multifunctional effects, TRAIP is involved in many other important signaling pathways (Bhat et al., 2018). The negative effect of TRAIP in TNF- induced NF-kB activation was introduced a few decades ago (Jackson and Bartek, 2009).

RAP80 (receptor-associated protein 80), a ubiquitin-binding protein of 719 amino acids, has two tandem ubiquitin-interacting domains that preferentially detect and bind to Lys-63-linked polyubiquitin chains, allowing the BRCA1-1 complex to reach regions of DNA damage (Besse et al., 2007, Kim et al., 2017). RAP80 localizes to sites of DNA damage to induce the DNA-damage response (DDR) (Besse et al., 2007). RAP80 plays a crucial role in maintaining genomic stability and tumor suppression (Kim et al., 2017, Kim et al., 2007). In DDR pathways, protein-protein interaction and post-translational modification have a key role in it (Besse et al., 2007, Lee et al., 2016). Ubiquitin-interacting motif (UIM) of RAP80 specifically recognizes Lys 63-linked histone ubiquitination, H2A, and H2AX, at sites of DNA damage. The translocation of the BRCA1-A complex to DNA-damage sites has been shown to regulate the G2/M checkpoint and DNA-damage repair and is required for cell survival (Lee et al., 2016, Lee and Choi, 1997, Nasreena et al., 2019). In our present study, different constructs of TRAIPCC, TRAIP CCLZ, TRAIP RINGCC and RAP80 Zn finger were designed to identify the best overexpression protein. An overexpressed and well-purified construct of TRAIPCC with corresponding amino acid 66–164 was used for *in vitro* study. Similarly, the well overexpressed and purified construct of the RAP80 zinc finger motif with corresponding amino acid 490-584was used for complex association formation. Our *in vitro* study shows that the TRAIPCC domain is critical for interaction with the RAP80 zinc finger motif and forms a complex with RAP80 Zinc finger motif. However, TRAIP RINGCC and TRAIP CCLZ of corresponding amino acid 1–157 and 66–260 failed to interact with the RAP80 zinc finger of corresponding amino acid 490–584 respectively. The purity and homogeneity of proteins were analyzed by SDS-PAGE gel. Furthermore, Size-exclusion chromatography confirmed the complex formation.

## 2 Results

### 2.1 TRAIP CC (66-164aa) and RAP 80 zinc finger (490–584 aa) forms a single homogenous trimeric peak

**Fig. 1:**
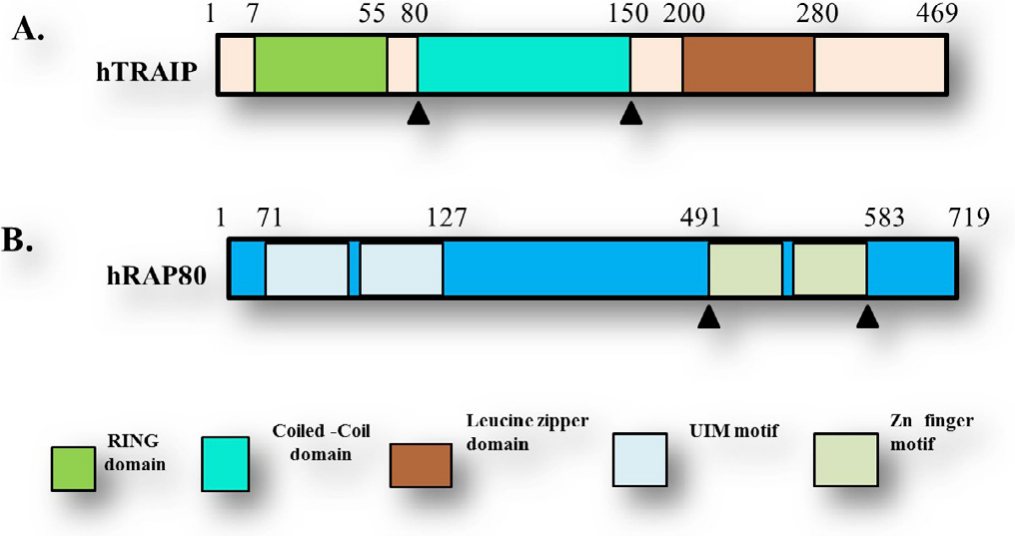
Schematics of TRAIP and RAP80. (A) The domain boundary of TRAIP with the number of amino acids from TRAIP RING, TRAIP CC, TRAIP Leucine zipper. (B) The domain boundary of RAP80 with the N-terminal end contains UIM motif and C-terminal side ZF motif shown.

The TRAIP (53 kDa) consists of 469 amino acids with an N-terminal RING motif followed by coiled-coil (CC) and leucine zipper (LZ) domain (Fig. 1A). The RING domain is known to possess E3 ubiquitin ligase activity (Bartek et al., 2007, O’Driscoll and Jeggo, 2006, Regamey et al., 2003). The corresponding amino acids 211–470 of TRAIP has been involved in a complex with CYLD and prevented the inhibitory activity of TRAIP (Su, 2006, Thompson and Schild, 2002). The RAP80 protein constituted 719 amino acids with the N-terminal UIM domain followed that two Zinc finger motif at the C-terminal end (Fig. 1B). RAP80 is a key signaling molecule in the DNA damage response (Wallace et al., 2014). C-terminal of RAP80 possesses a Zinc finger motif that has been known to interact with TRAIP CC protein (Besse et al., 2007, Wang et al., 2007).

**Fig. 2:**
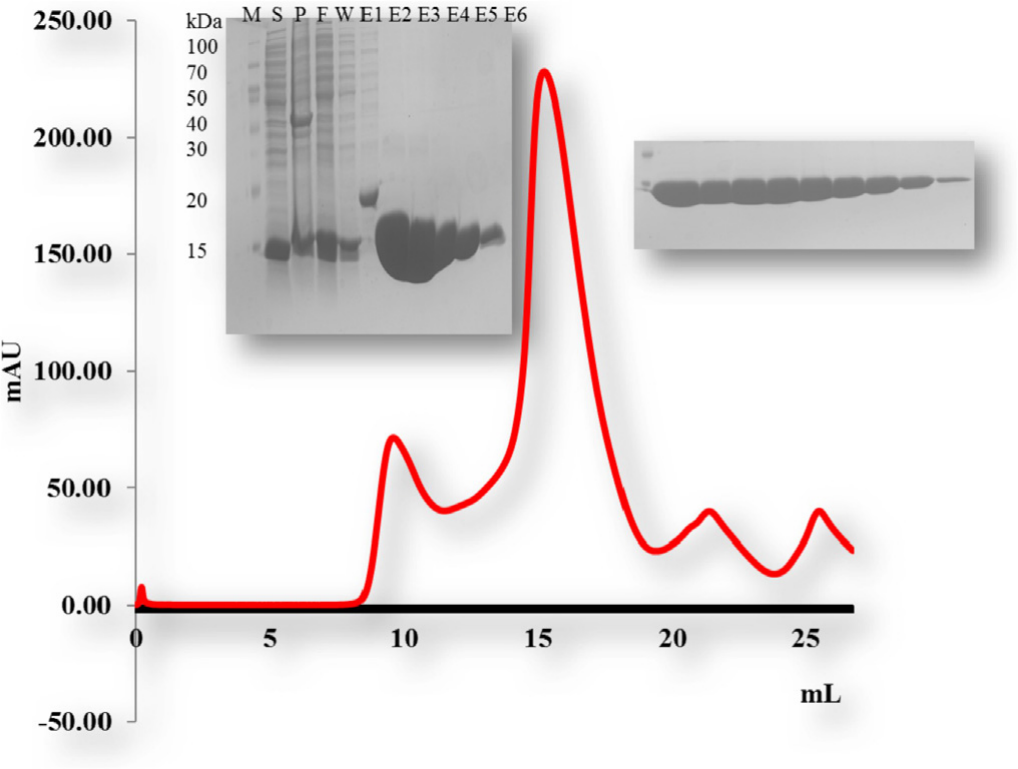
Gel filtration chromatogram. His tag and gel filtration chromatography of TRAIPCC (66–164 aa) domain. The SDS-PAGE judged purity of both Ni-affinity and purified fractions of gel filtration chromatography. M# marker, S# supernatant, P# pellet, F# flow through, W# wash and E1-E6 (Elution).

**Fig. 3:**
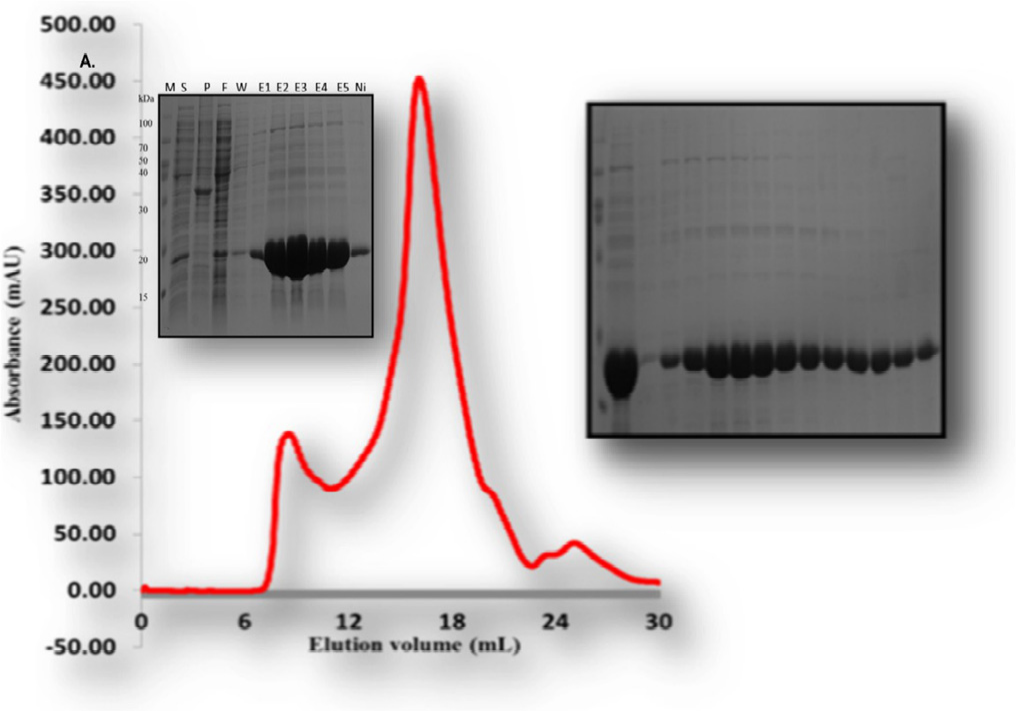
Gel filtration chromatogram. His tag and gel filtration chromatography of TRAIP RINGCC (1-157aa) domain. The SDS-PAGE judged purity of both Ni-affinity and purified fractions of gel filtration chromatography. M# marker, S# supernatant, P# pellet, F# flow through, W# wash and E1-E5 (Elution).

**Fig. 4:**
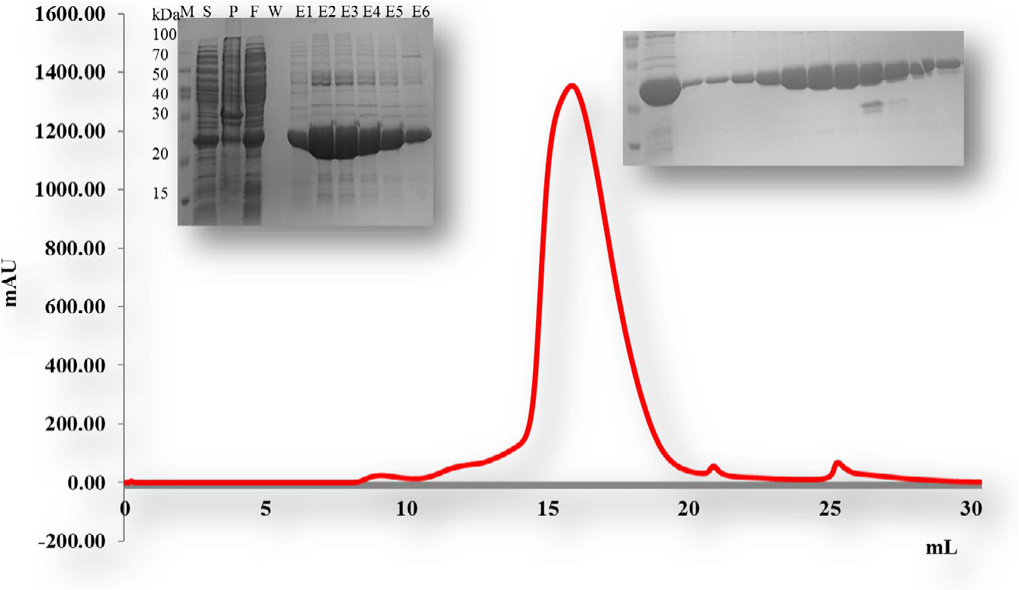
Gel filtration chromatogram. His tag and gel filtration chromatography of TRAIPCCLZ (66–280 a.a) domain with SDS-PAGE judged homogeneity of a protein. The Ni-affinity fractions show on the left side of the peak. A single peak was observed around 17 ml of elution volume. The purified fractions of main peak were loaded on SDS-PAGE to analyze protein purity as shown on the right side of peak. M# marker, S# supernatant, P# pellet, F# flow through, W# wash and E1-E6 (Elution).

**Fig. 5:**
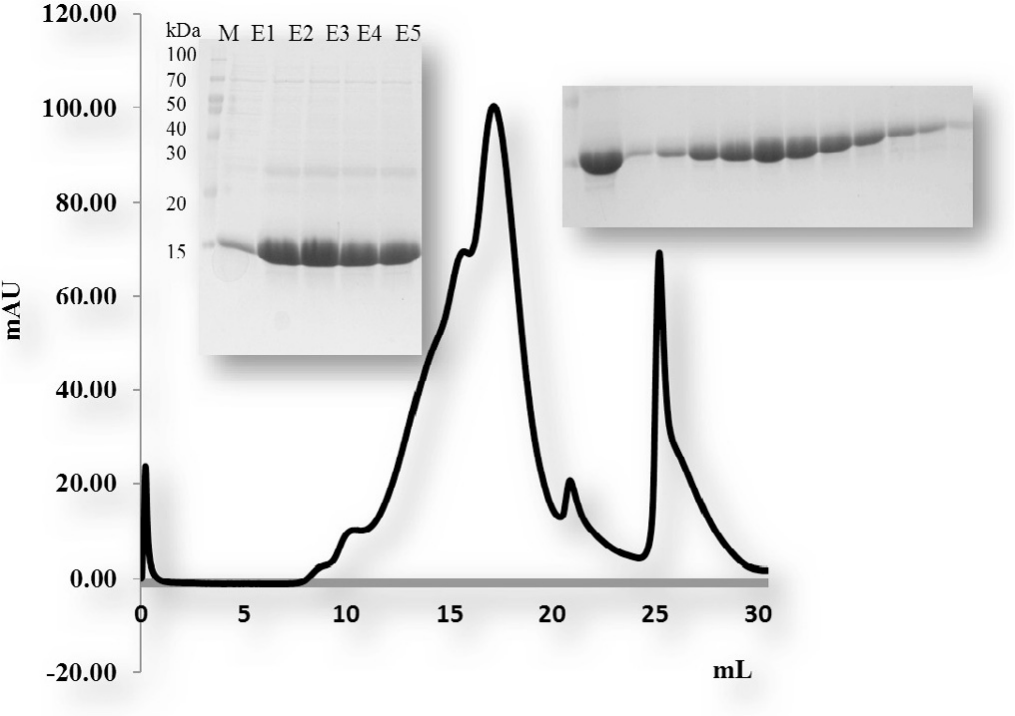
Gel filtration chromatogram. His tag and gel filtration chromatography of the RAP80 Zn finger motif (66–164 a.a) domain. A single peak of RAP80 Zn finger motif was eluted around 17 ml in SEC. Both Ni-affinity and purified fractions of gel filtration chromatography with SDS-PAGE shows on the top of the figure.M #marker and E1-E5 (Elution).

For biochemical studies *in vitro*, we produced the overexpression construct of each protein *viz*, TRAIP CC (66–164 aa), TRAIP RINGCC (1–517), TRAIP CCLZ (60–280 aa) and RAP 80 (490–584 aa). Each of one protein was overexpressed and purified by Ni affinity followed by size exclusion chromatography. The size exclusion chromatography showed that the TRAIP CC domain of corresponding amino acid 66–164 was eluted at approximately 17 ml (Fig. 2). The gel filtration chromatography of the TRAIP RINGCC was eluted between 9 and 18 ml which shows RING mediated oligomerization of CC domain (Fig. 3). The gel filtration chromatography of the CCLZ domain was eluted at 17 ml (Fig. 4). The size exclusion chromatography of RAP 80 zinc-finger of corresponding amino acid 490–584 showed that it was eluted at 17 ml which suggests the trimeric in solution (Fig. 5).

**Fig. 6:**
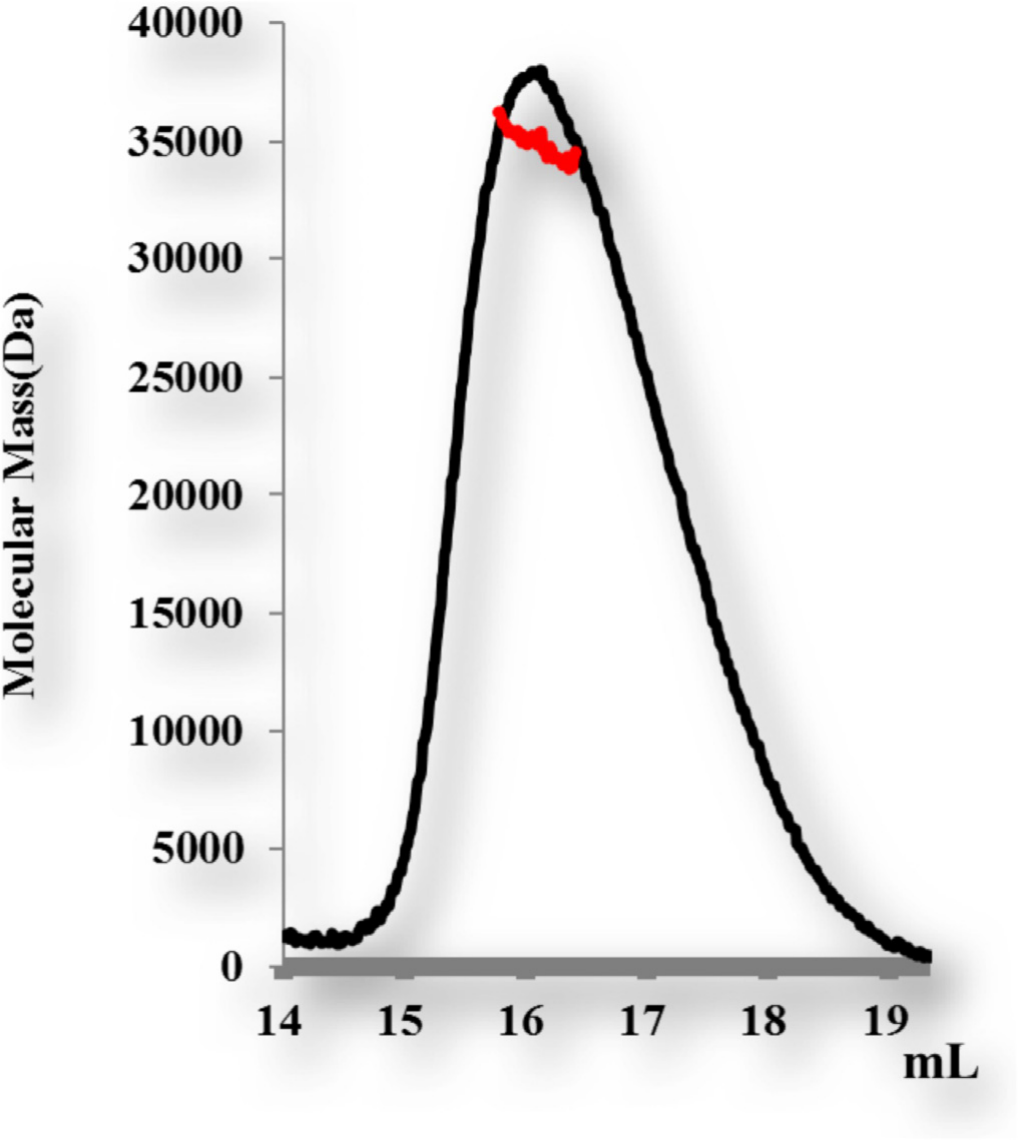
MALS result of TRAIP coiled coil domain of corresponding amino acid 66–164 with 0.9% fitting error.

To analyze the stoichiometry of TRAIP CC of corresponding amino acid 66–164 in solution by calculating absolute molecular mass, we performed analysis through MALS. The C terminal containing Hexa His tag of TRAIP CC of corresponding amino acid 66–164 with calculated molecular weight 12325 Da, and the major molecular weight 34, 254 Da (0.9% fitting error) based on our MALS result, with a polydispersity of 1.012 as shown in (Fig. 6). The results of size exclusion chromatography and MALS showed TRAIP CC is a trimer in solution.

To investigate the complex assembly of TRAIP CC (66–164 aa), TRAIP RINGCC and TRAIP CCLZ (60–280 aa) with RAP 80 zinc finger (490–584 aa) using structural and biochemical assays *in vitro*, we designed different constructs of TRAIP CC domain shows in Table 1, which is known to interact with the RAP 80 containing zinc finger domain (490–584 aa). TRAIP RINGCC as shown in Table 2 and TRAIP CCLZ as shown in Table 3 has also designed different constructs to find the best-overexpressed construct. Similarly, different constructs of RAP80 Zn finger motif was designed as shown in Table 4. The gel chromatography profile revealed that TRAIP CC (66–164 aa) domain eluted at 17 ml, indicating the formation of a trimer as reported previously in a different construct of protein by (Bhat et al., 2018). This is in accordance with previous findings.

**Table 1:** Different constructs of TRAIP coiled-coil domain protein.

Name	Species	Region	Amino acid	DNA	Enzyme	Vector	PCR	Cloning	expression
TRAIP-1	Human	66(L)-164(K)	99 a.a	297 bp	NdeI/XhoI	pET24a	successful	successful	over expressed
TRAIP-2	Human	72(N)-167(E)	96 a.a	288 bp	NdeI/XhoI	pET24a	successful	successful	expressed

**Table 2:** Different constructs of TRAIP RINGCC domain protein.

Name	Species	Region	Amino acid	DNA	Enzyme	vector	PCR	Cloning	expression
TRAIP-3	Human	1(M)-150(K)	150 a. a.	450 bp	NdeI/XhoI	pET24a/pOKD	successful	successful	No expression
TRAIP-4	Human	1(M)-154(E)	154 a. a.	462 bp	NdeI/XhoI	pET24a/pOKD	successful	successful	No expression
TRAIP-5	Human	1(M)-157(R)	157 a. a.	471 bp	NdeI/XhoI	pET24a/pOKD	successful	successful	Over Expression
TRAIP-6	Human	1(M)-162(K)	162 a. a.	486 bp	NdeI/XhoI	pET24a/pOKD	successful	successful	No expression

**Table 3:** Different constructs of TRAIP coiled-coil Leucine Zipper domain protein.

Name	Species	Region	Amino acid	DNA	Enzyme	Vector	PCR	Cloning	Expression
TRAIP-7	Human	66(L)-280(L)	215a.a	645 bp	NdeI/XhoI	Pokd	successful	successful	over expressed
TRAIP-8	Human	66(L)-276(E)	211a.a	633 bp	NdeI/XhoI	pET24a	successful	successful	expressed
TRAIP-9	Human	66(L)-272(T)	207a.a	621 bp	NdeI/XhoI	Pokd	successful	successful	expressed

**Table 4:** Different constructs of RAP80 Zinc finger motif.

Name	Species	Region	Amino acid	DNA	Enzyme	Vector	PCR	Cloning	Expression
RAP80-1	Human	490(K)-584(Q)	95a.a	285 bp	NdeI/XhoI	pET24a/pOKD	successful	successful	over expressed
RAP80-2	Human	490(K)-590(Q)	101a.a	303 bp	NdeI/XhoI	pET24a/pOKD	successful	successful	expression
RAP80-3	Human	496(T)-584(Q)	89a.a	267 bp	NdeI/XhoI	pET24a/pOKD	successful	successful	expression
RAP80-4	Human	496(T)-584(Q)	95a.a	285 bp	NdeI/XhoI	pET24a/pOKD	successful	successful	expression

**Fig. 7:**
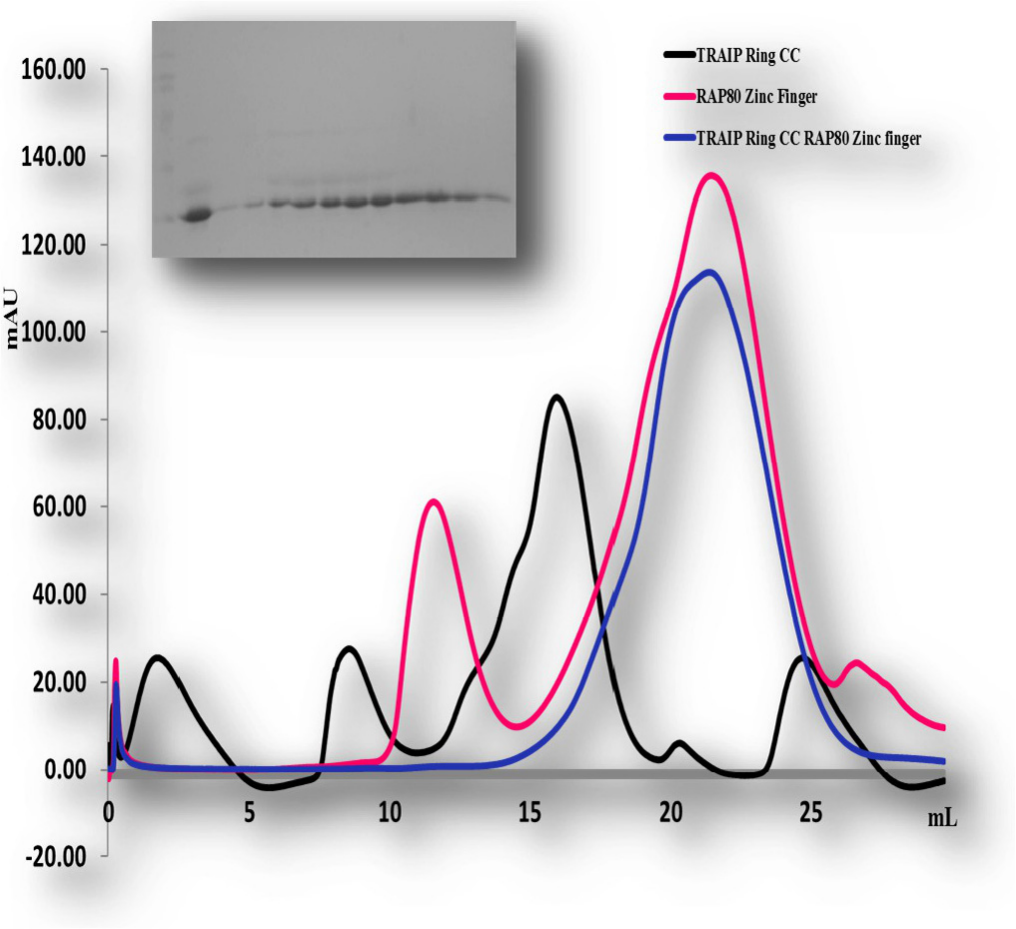
Gel filtration chromatogram. Size-exclusion chromatography (SEC) profiles of TRAIP RINGCC and RAP80 Zn finger *in vitro*. TRAIP RINGCC domain did not interact with the RAP80Zn finger *in vitro*.

**Fig. 8:**
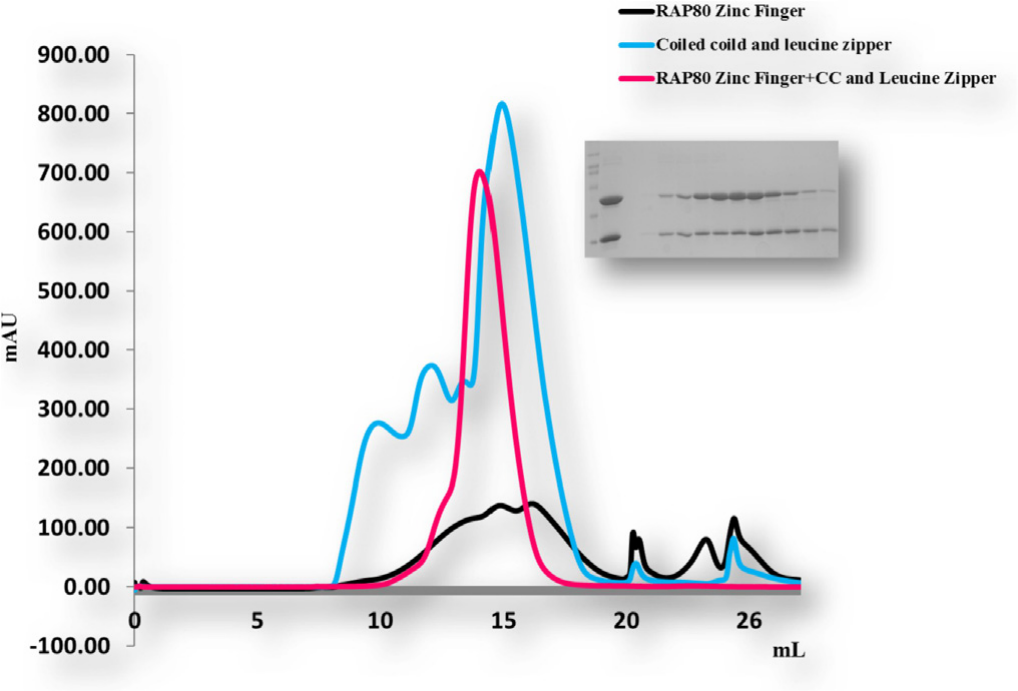
Gel filtration chromatogram. Size-exclusion chromatography (SEC) profiles of TRAIP CCLZ and RAP80 Zn finger *in vitro*. TRAIPCCLZ domain failed to interact with the RAP80Zn finger *in vitro*.

**Fig. 9:**
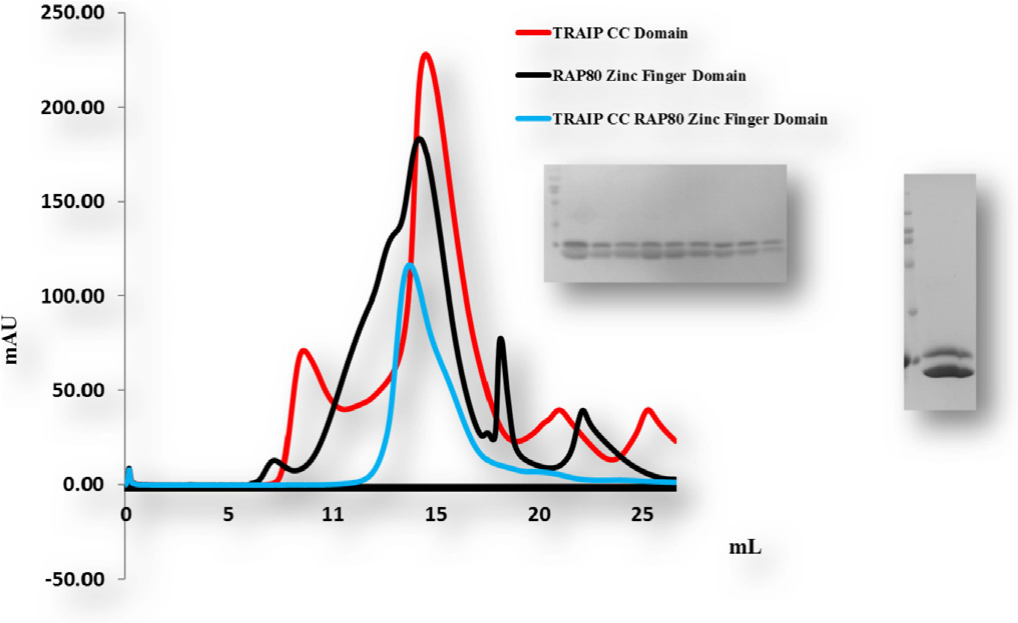
Gel filtration chromatogram. Size-exclusion chromatography (SEC) profile of TRAIP CC interaction with specifically RAP80 Zn finger *in vitro*. SDS-PAGE on the right side of the peak shows two bands of melted protein crystalized complex loaded on SDS-PAGE.

To confirm these results we performed size exclusion chromatography. The mixtures of different combination did not produce complex peaks (Fig. 7, Fig. 8). Only the mixture of TRAIP CC of corresponding amino acid 66–164 and RAP 80 zinc-finger of corresponding amino acid 490–584 co-migrated in SDS PAGE (Fig. 9). This result showed consistency with our previous results, revealing that TRAIP CC (66–164 aa) specifically binds with RAP 80 zinc finger (490–584 aa) *in vitro*.

## 3 Discussion

Human TRAIP is constituted of 469 amino acids with putative domains, include an N-terminal RING domain followed by coiled-coil (CC) and leucine zipper (LZ) domains (Almeida et al., 2011, Bartek et al., 2007). The RING domain has been detected in many E3 ligases and is critical for the activity of ubiquitin ligation. TRAIP protein has E3 ubiquitin ligase activity to TANK-binding kinase 1 has been shown (Wu et al., 2012). TRAIP with corresponding residues 211–470 at the C-terminal known to be interacted directly with CYLD and enhanced the inhibitory activity of TRAIP. TRAIP marks a crucial signaling molecule, implicated with several signaling pathways (Bhat et al., 2018).

A ubiquitin-binding protein, RAP80 (receptor-associated protein 80) is constituted of 719 amino acid is a novel binding partner of Human TRAIPCC proteins (Besse et al., 2007), contains two tandem ubiquitin-interacting motifs that specifically recognize and bind to Lys-63-linked polyubiquitin chains, allows BRCA1-1 complex to sites of DNA damage (Besse et al., 2007, Kim et al., 2017). RAP80 localizes to sites of DNA damage to induce the DNA-damage response (DDR) and plays a crucial role in maintaining genomic stability and tumor suppression (Besse et al., 2007, Kim et al., 2017, Kim et al., 2007). During normal cellular processes, such as replication, it is possible as result DNA lesions occur or prone to various environmental hazards such as ionizing radiation and ultraviolet light (Yan and Jetten, 2008). To prevent the accumulation of DNA insults and genomic integrity or damaged genetic transmission from mother to daughter cells, DNA damage repair mechanisms with sophisticated cell cycle checkpoint pathways are developed (Yan et al, 2007, Yin et al., 2012). The DNA double-strand break (DSB), most deleterious type of DNA damage among various kinds of DNA insults, and subsequently alters genomic stability and induces tumorigenesis. DNA damage response factors (DDR) are frequently recruited at DNA damage and activates cell cycle checkpoints and DNA repair mechanisms (Yoon et al., 2014). Subsequently, genomic instability and tumorigenesis are caused by the loss of these DNA damage response factors (Zhang et al., 2012, Zhang et al., 2012). Large multi subunit protein complexes are often formed by these DNA damage response factors. Overall, the interaction between TRAIPCC and RAP80 Zn finger was observed *in vitro*, the TRAIPCC region is critical for an association of the complex with the RAP80-Zn finger motif. This interaction approach may be important for translocation of RAP80 to the sites of DNA lesions. Our biochemical characterization reveals that the N-terminal coiled-coil (CC) domain is crucial for interaction with RAP80 Zn finger domain protein.

## 4 Materials and methods

### 4.1 TRAIP CC, TRAIP RINGCC, TRAIP CCLZ, and RAP80 Zinc finger cloning, expression and purification

The full length of Human cDNA TRAIP (1–280 amino acids) and Human RAP80 (1–719 amino acids) were used as a template in the Polymerase chain reaction. The PCR products were digested by NdeI and XhoI restriction enzymes (Enzynomics). Consequently, the pET24a plasmid was digested with the same enzymes. The overexpressed construct of TRAIPCC corresponding amino acid 66–164, TRAIP RINGCC of corresponding amino acid 1–157, TRAIP CCLZ corresponding amino acid 60–280 and RAP80 Zinc finger corresponding amino acid 490–584 were then sub-cloned into plasmid vector pET24a with His-tag His6 at C-terminal purchased from Novagen. Plasmids were then separately transformed into BL21 cell (DE3) competent cells and then speckled on Luria-Bertani (LB) agar plates containing the appropriate antibiotics (60ul/ml). Consequently, the plates were incubated at 37 °C until the single colony grows bigger. Each colony from three different constructs were picked and inoculated into the 10 ml Luria broth medium followed by overnight incubation at 37 °C shaking incubator. The 3 ml of the pre-inoculated medium was then inoculated into a 1000 ml LB medium with appropriate antibiotics. The cells were growing until the O_D_ reaches between 0.6 and 0.7 nm, which was checked at 600 nm. A 0.25 mM isopropyl-β-D thiogalactopyranoside (IPTG) was induced in the medium and was incubated at 20 °C overnight. The bacteria expressing protein was pelleted down by centrifugation at 3500 rpm for 15 min. The cells were then sonicated in 40 ml of lysis buffer, supplemented with phenyl methane sulfonyl fluoride (PMSF). Subsequently, the cell lysate was centrifuged at 16000 rpm for 30 min. The supernatant was collected and subjected to Ni-NTA affinity column for the separation of protein. Furthermore, the unwanted proteins were removed by 50 ml of washing buffer and subsequently the protein of interest was eluted with a high concentration of imidazole. A 0.5 ml fraction of the target protein being collected over a total of 2 ml. The homogeneity of each protein was more than 80% which was analyzed by SDS-PAGE gel. TRAIP CC (66–164 aa), TRAIP RINGCC (1-157aa), TRAIP CCLZ (60-280aa) and RAP 80 zinc finger (490–584 aa) proteins were collected and combined up to 2 ml. Each protein was loaded on size chromatography column HR 10/30 of superdex 200 that was pre-equilibrated with a solution of 20 mM Tris-HCl at pH8.0 and 150 mM NaCl. The fractions of the main peak of TRAIP CC (66–164 aa), TRAIP RINGCC (1-157aa), TRAIP CCLZ (60–280 aa) and RAP 80 zinc finger (490–584 aa) were pooled and stored 4 °C for further characterization. Cloning, protein expression, and purification of the human TRAIP domains was conducted as described previously (Zhou and Geahlen, 2009).

### 4.2 MALs

The protein TRAIP CC corresponding amino acid 66–164 was used to assess the absolute molecular mass by multi-angle light scattering (MALS). TRAIPCC was purified by two rapid steps, Ni-affinity chromatography, and size exclusion chromatography column HR 10/30. The main peak fractions of the purified TRAIP CC were collected. Centrifugation (14000 rpm) was done at 4 °C for 10 min to remove the precipitate before loading on size exclusion chromatography column HR 10/30 (bed dimensions 10* 300 mm), which was pre-equilibrated with a solution containing 20 mM Tris-HCl at pH 8 and 150 mM NaCl. Moreover, the system was connected with three-angle light scattering refractive index detector and mini-DAWN treos MLAS detector (Wyatt Technology, Santa Barbara, CA, USA). After every 0.5 s, the data collected was analyzed by the ASTRA program, suggesting molar mass plus mass distribution of each sample.

### 4.3 Protein complex assay by size-exclusion chromatography

Purified protein samples of TRAIP CC of corresponding amino acid 66–164, TRAIP CCLZ of corresponding amino acid 60–280 and RAP 80 zinc-finger of corresponding amino acid 490–584 from Ni affinity chromatography were mixed in a molar ratio of approximately 1:1 and pre-incubated for 30 min at 4 °C. The protein mixture was passed through a superdex 200 gel filtration column HR 10/30 (GE health care) which was pre-equilibrated with a solution containing 20 mM Tris-HCl at pH8.0 and 150mMNaCl [31].

Declaration

## 5 Ethics approval and consent to participate

This article does not contain any studies with human participants or animals performed by any of the authors.

## 6 Availability of data and materials

The datasets used in the current study are available from publicly.

## Declaration of Competing Interest

The authors declare that they have no known competing financial interests or personal relationships that could have appeared to influence the work reported in this paper.
