# Supplementary material for: In vitro assembly complex formation of TRAIP CC and RAP 80 zinc finger motif revealed by our study
Source: Saudi J Biol Sci. 2021 Aug 30;28(12):7511–6. doi: 10.1016/j.sjbs.2021.08.083 (PMC8626312; doi:10.1016/j.sjbs.2021.08.083)
Supplement: Supplementary data 1 [file mmc1.pptx]

## Slide 1
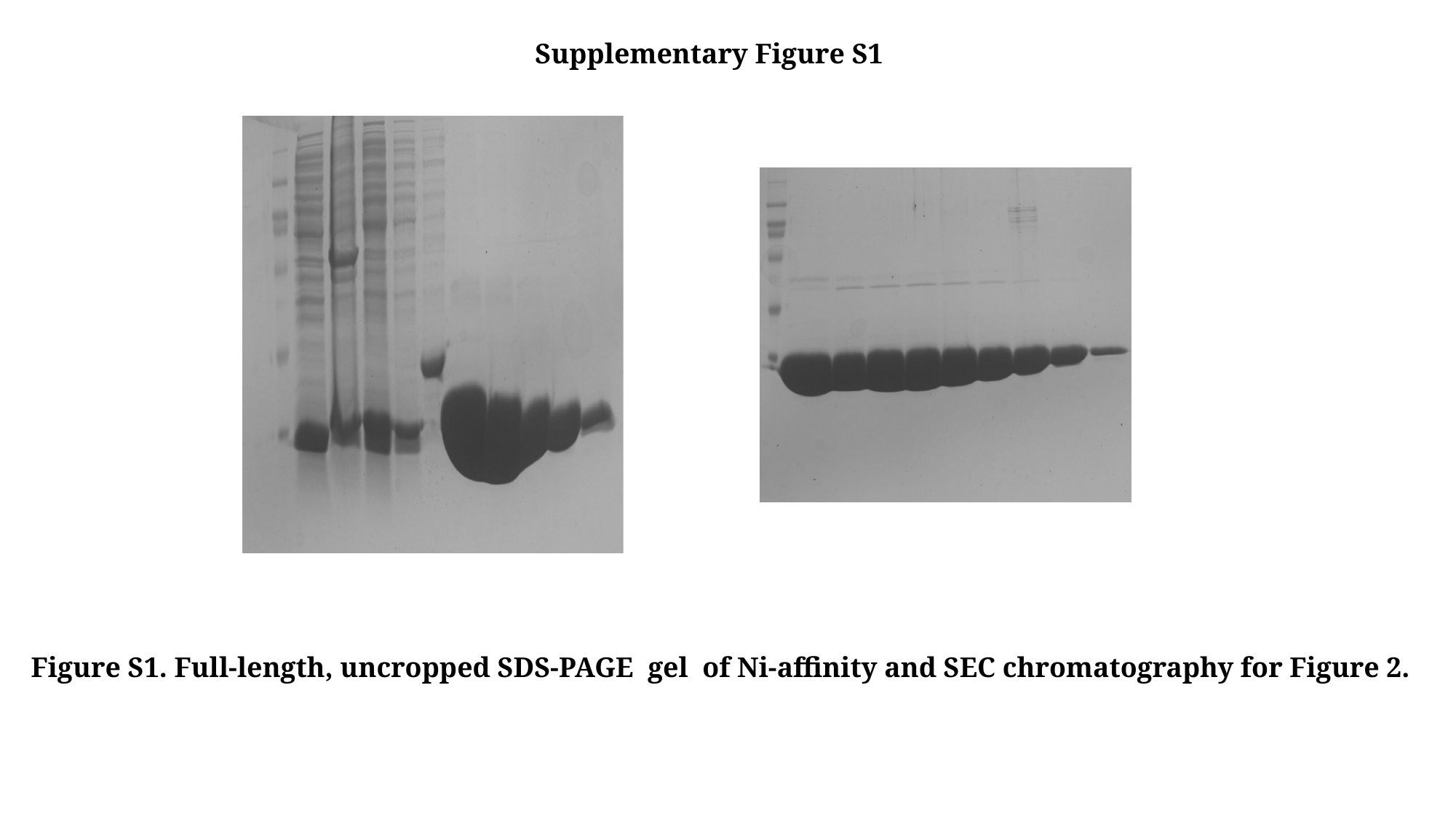

Supplementary Figure S1
Figure S1. Full-length, uncropped SDS-PAGE gel of Ni-affinity and SEC chromatography for Figure 2.

## Slide 2
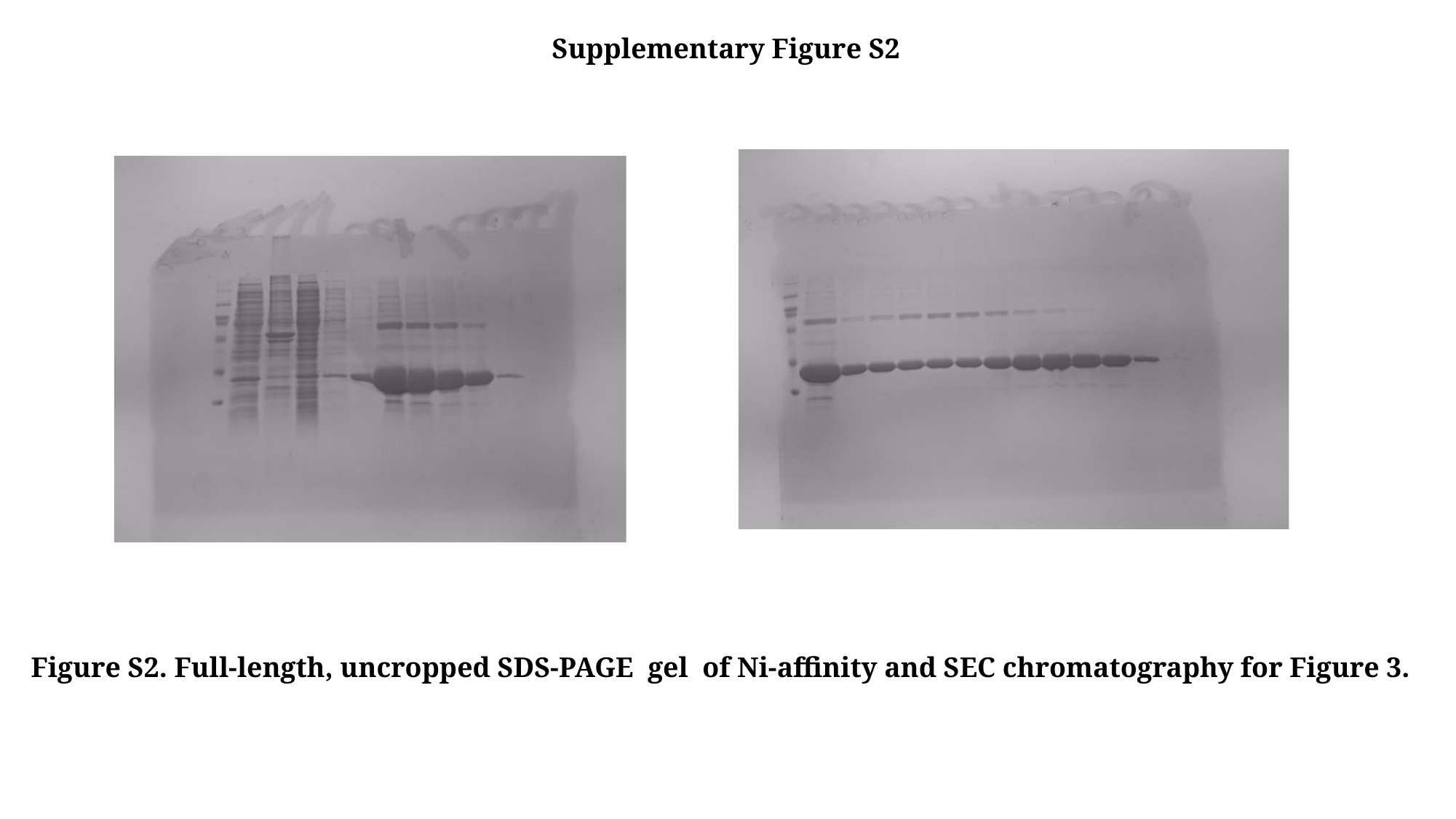

Supplementary Figure S2
Figure S2. Full-length, uncropped SDS-PAGE gel of Ni-affinity and SEC chromatography for Figure 3.

## Slide 3
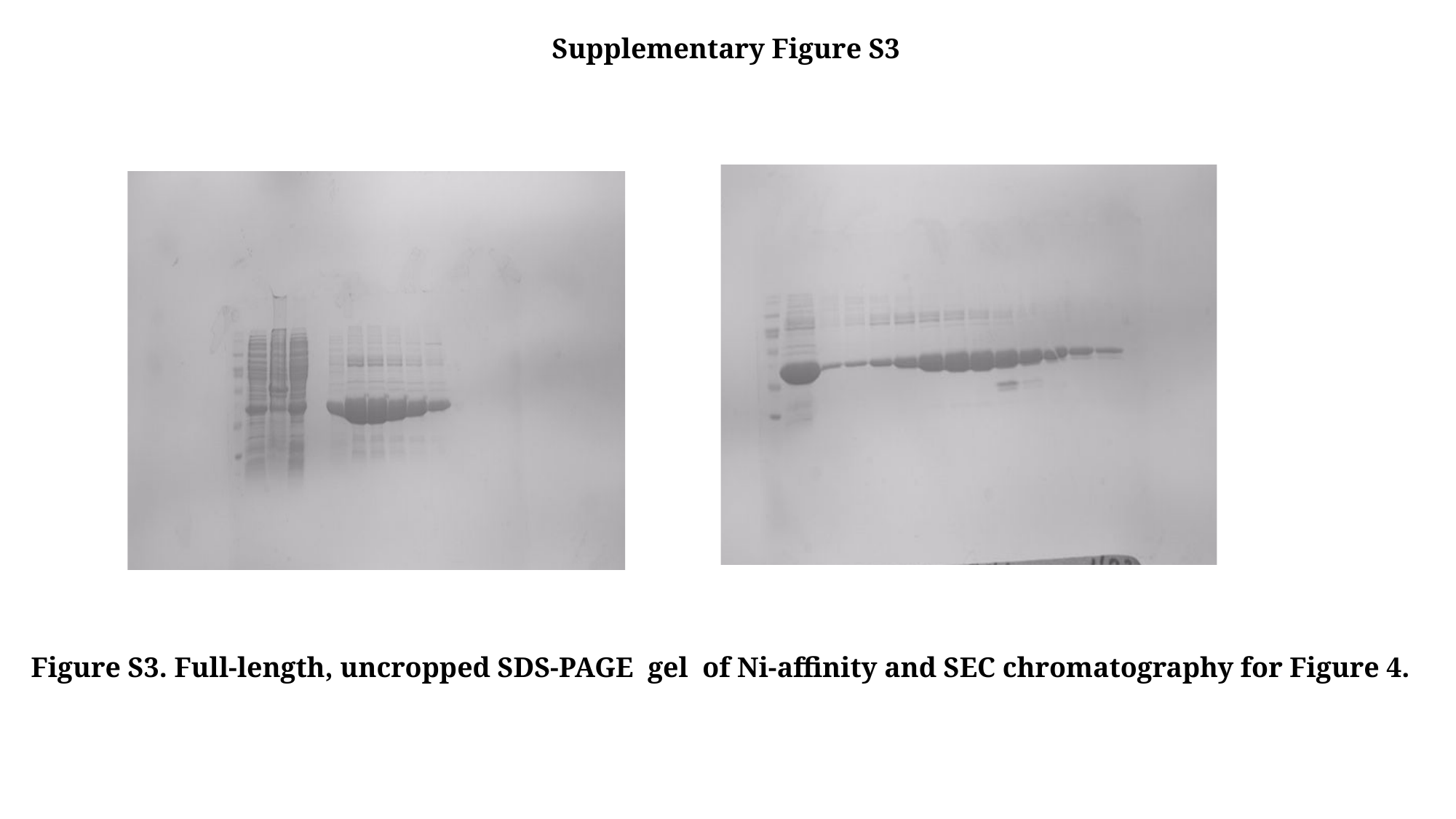

Supplementary Figure S3
Figure S3. Full-length, uncropped SDS-PAGE gel of Ni-affinity and SEC chromatography for Figure 4.

## Slide 4
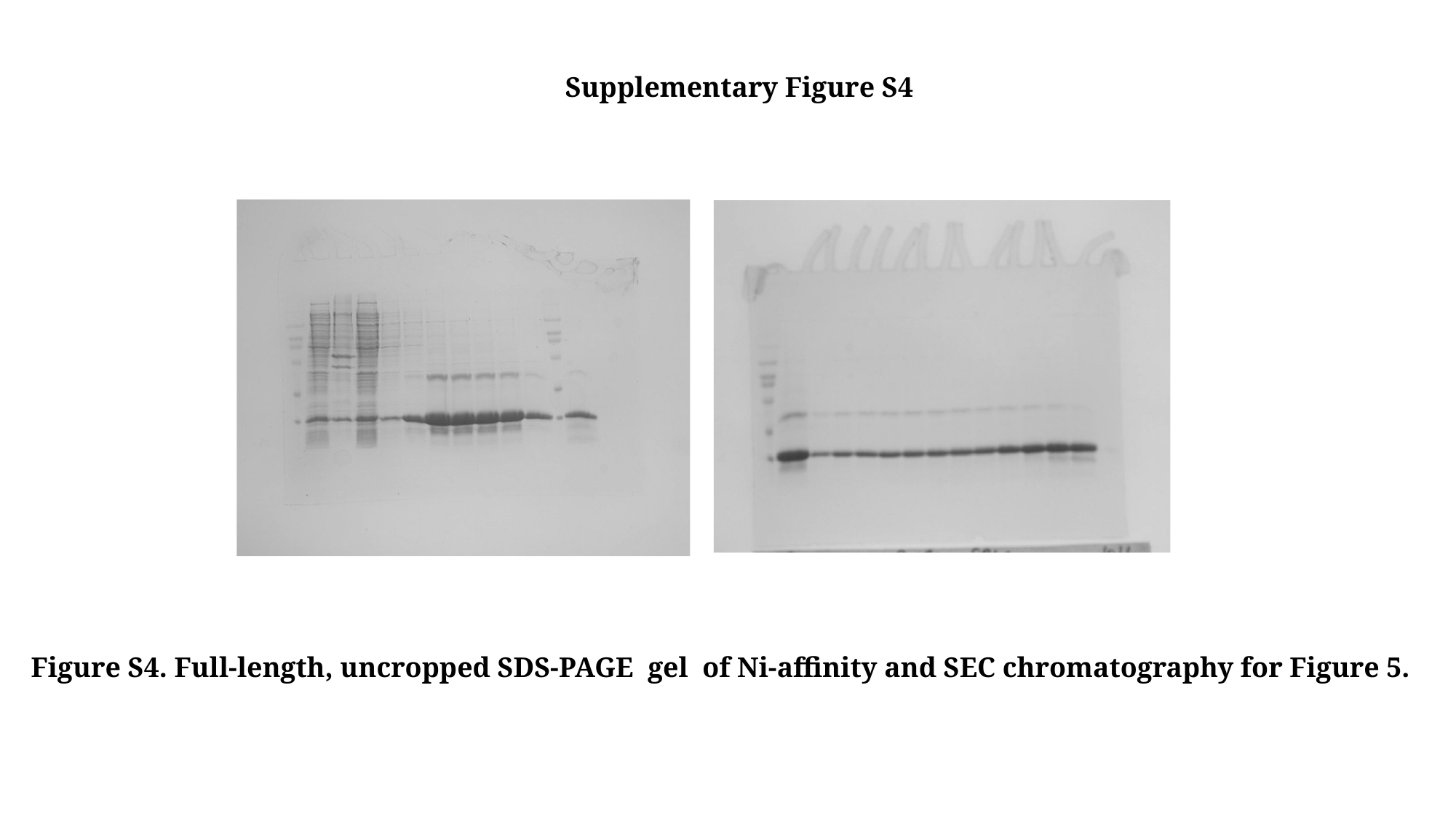

Supplementary Figure S4
Figure S4. Full-length, uncropped SDS-PAGE gel of Ni-affinity and SEC chromatography for Figure 5.

## Slide 5
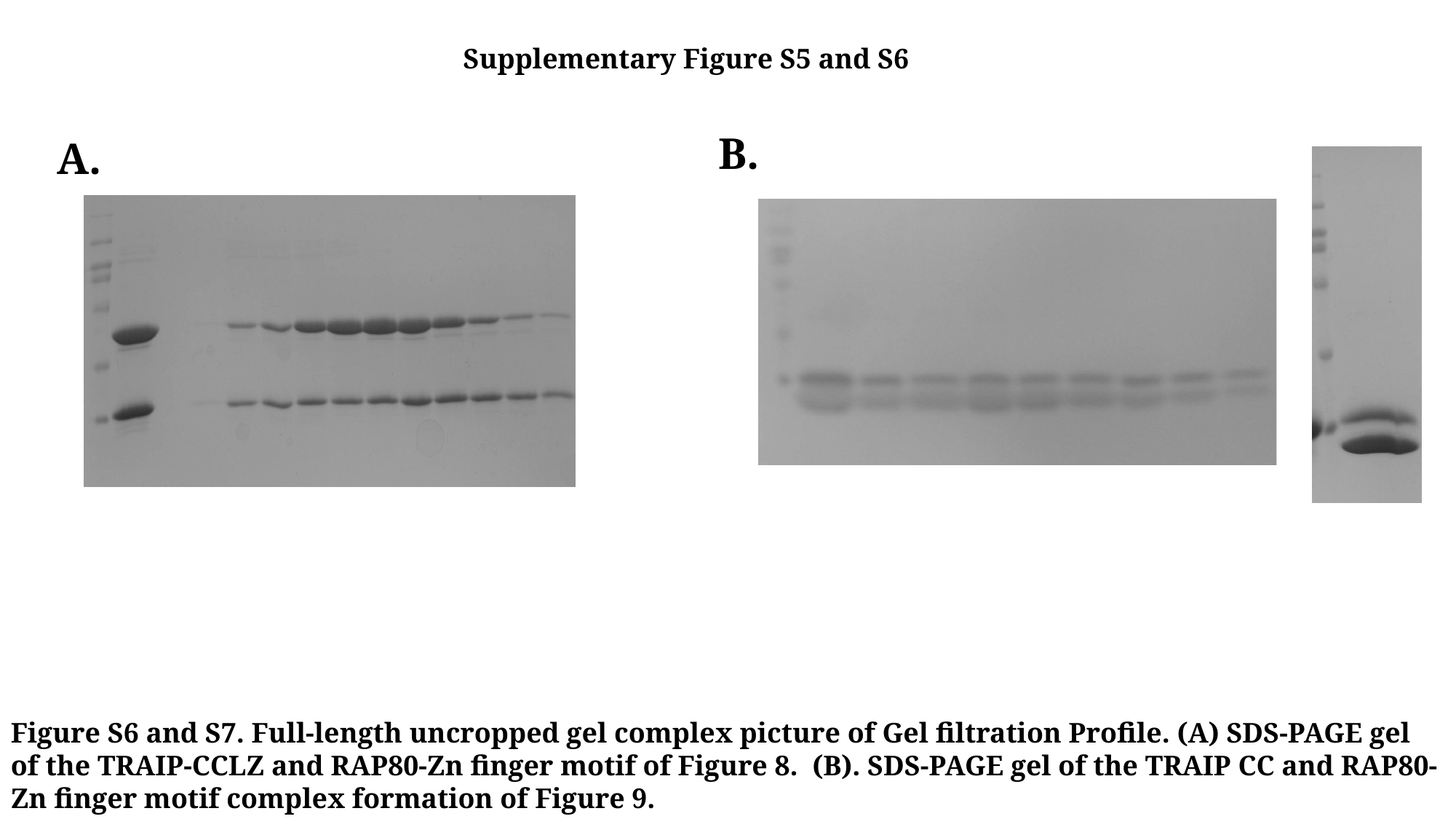

Supplementary Figure S5 and S6
B.
A.
Figure S6 and S7. Full-length uncropped gel complex picture of Gel filtration Profile. (A) SDS-PAGE gel of the TRAIP-CCLZ and RAP80-Zn finger motif of Figure 8. (B). SDS-PAGE gel of the TRAIP CC and RAP80-Zn finger motif complex formation of Figure 9.
